# Optimal timing of surgical antimicrobial prophylaxis in laparoscopic surgery: a before-after study

**DOI:** 10.1186/s13756-018-0424-z

**Published:** 2018-10-31

**Authors:** Akane Takamatsu, Yasuaki Tagashira, Kaori Ishii, Yasuhiro Morita, Yasuharu Tokuda, Hitoshi Honda

**Affiliations:** 1 Division of Infectious Diseases, Tokyo Metropolitan Tama Medical Center, 2-8-29 Musashidai, Fuchu, Tokyo, Japan; 2 Department of Infection Control, Tokyo Metropolitan Tama Medical Center, 2-8-29 Musashidai, Fuchu, Tokyo, Japan; 3 Department of Surgery, Tokyo Metropolitan Tama Medical Center, 2-8-29 Musashidai, Fuchu, Tokyo, Japan; 4 Muribushi Project for Teaching Hospitals, 3-42-8-901 Iso, Urasoe, Okinawa, Japan

**Keywords:** Surgical antimicrobial prophylaxis, Laparoscopic surgery, Surgical site infection

## Abstract

**Background:**

The optimal timing of preoperative surgical antimicrobial prophylaxis (SAP) remains uncertain. This study aimed to evaluate the impact of changing the timing of SAP on the incidence of surgical site infection (SSI) in laparoscopic surgery.

**Methods:**

We performed a before-after study from August 2014 through June 2017 to assess the impact of changes in the timing of SAP on the incidence of SSI at a 790-bed tertiary care center in Japan. The intervention was the administration of SAP immediately after the study patients entered the operating room for laparoscopic surgery.

**Results:**

In total, 1397 patients who met the inclusion criteria were analyzed. After the intervention, the median time between the time of SAP completion and the time of surgical incision changed from 8 min to 26 min (*p* <  0.001), and the number of cases without SAP completion prior to surgical incision decreased (16.8% vs. 1.8%; *p* <  0.001). However, changes in the overall incidence of SSI did not significantly differ between the pre-intervention and the intervention groups (13.8% vs. 13.2%; *p* = 0.80).

**Conclusions:**

Although the timing of preoperative SAP improved, the intervention did not have a significant impact on reducing the incidence of SSI in the current study. Besides preoperative SAP, multidisciplinary approaches should be incorporated into projects aimed at comprehensively improving surgical quality to reduce SSI.

## Introduction

Surgical site infection (SSI) is one of the most common healthcare-associated infections in the acute care setting [1]. The incidence of SSI varies sociogeographically and by patient-related factors, such as co-morbidities and surgery-related factors, including type of surgery [1, 2].

Surgical antimicrobial prophylaxis (SAP) is one of the most important modifiable factors for reducing SSI incidence. Administering SAP within 120 min before surgery decreased the risk of SSI, and the rate of SSI increased with each hour after the incision until SAP was administered [3]. Guidelines for preventing SSI were published by the World Health Organization (WHO) in 2016 and by the Centers for Disease Control and Prevention (CDC) in 2017 [1, 4]. Although these guidelines recommend preoperative SAP within one hour of surgical incision, the optimal timing of SAP during this period (e.g., 0–30 min versus 30–60 min before incision) remains unclear.

In Japan, a number of studies on the incidence of SSI and risk factors associated with SSI in selected surgical procedures were done using a Japanese national database [5, 6]. The cumulative incidence of SSI in gastric and colorectal surgery ranged from 8.8 to 17.8% [5, 6]. Moreover, cases without complete preoperative SAP prior to the first incision in laparoscopic surgery were occasionally observed at the study institution. The infection control team at the study institution administered preoperative SAP earlier to ensure adequate time between the completion of SAP and the first skin incision as a part of a standard quality improvement initiative. The aim of this study was to evaluate the impact of changing the timing of preoperative SAP on SSI incidence in laparoscopic surgery.

## Methods

### Study design and setting

This before-after study was conducted from August 2014 to June 2017 at Tokyo Metropolitan Tama Medical Center, a 790-bed tertiary care center in Tokyo. Approximately 7000 patients annually undergo surgery at the study institution. The patients’ informed consent was obtained before surgery. The institutional review board at Tokyo Metropolitan Tama Medical Center approved this study.

### Participants

Patients over age 16 years who underwent any form of laparoscopic surgery including cholecystectomies, colectomies, gastrectomies, appendectomies, and proctectomies at the study institution were included for analysis. Patients who underwent laparoscopic surgery without follow-up information 30 days after the procedure were excluded.

### Intervention

During the pre-intervention period (August 2014 – June 2016), the timing of preoperative SAP differed for each operation. In July 2016, the practice of administering SAP immediately after patients enter the operating room was implemented. Once patients were transferred into the operating room, intravenous SAP was immediately administered by anesthesiologists. If vancomycin or fluoroquinolone was used for SAP, administration of the agent was begun in the hospital ward before the patient was transferred to the operating room because both of these agents require a longer period of administration. Besides the intervention noted above, there were no concurrent changes in perioperative management, including the type and dosage of antimicrobials for SAP during the study period. The recommended SAP at the study institution is described in Appendix 1.

### Data collection

Clinical data were extracted from the electronic medical records. We also determined the type of laparoscopic surgery done, time of administration and completion of preoperative SAP, the American Society of Anesthesiologists (ASA) score, wound class, demographic characteristics, and underlying illnesses. Because SAP was administered intravenously over 15–30 min, we collected the data on the time of SAP completion and surgical incision. The primary outcome was the incidence of SSI as defined by the CDC criteria [7, 8]. Nurses and physicians in the department of infection control determined whether SSI developed in the study subjects. Patients who were discharged within the follow-up period were observed at follow-up visits as outpatients. The primary interest of this study was to examine if changing the timing of preoperative SAP affected the incidence of SSI.

### Statistical analysis

We compared the incidence of SSI between the pre-intervention and intervention periods using the chi-square test. We also compared other, selected factors between the study periods. The changes in SSI incidence due to the intervention were also evaluated by segmented regression analysis of interrupted time series (ITS) data. For the sensitivity analysis, factors associated with SSI in the study period were also investigated. The usual time point of SAP (i.e., 31–60 min prior to surgical incision as a reference, in comparison with 0–30 min and > 60 min) was used as a variable in the final model. Categorical variables were compared using Fisher’s exact test or the chi-square test as appropriate. Continuous variables were compared using the Mann-Whitney *U* test. All the analyses were performed using Stata version 15 (StataCorp, College Station, TX, USA) and SPSS version 21 (IBM, Armonk, NY, USA).

## Results

**Fig. 1:**
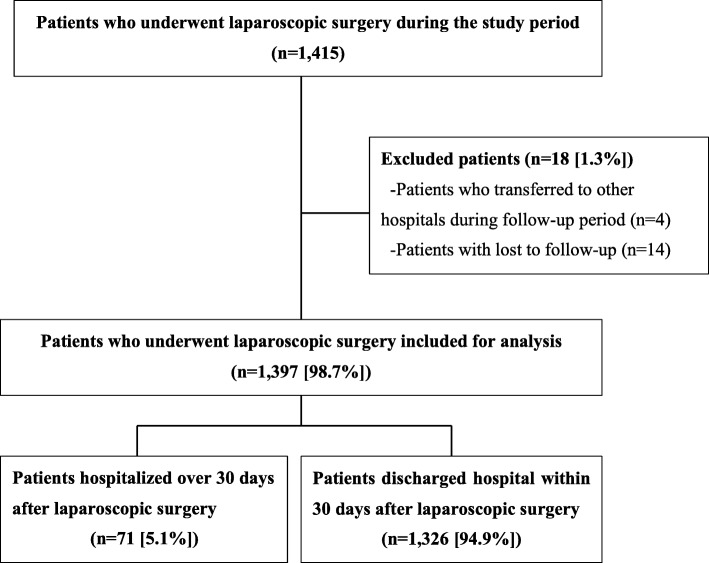
Description of the study population

**Fig. 2:**
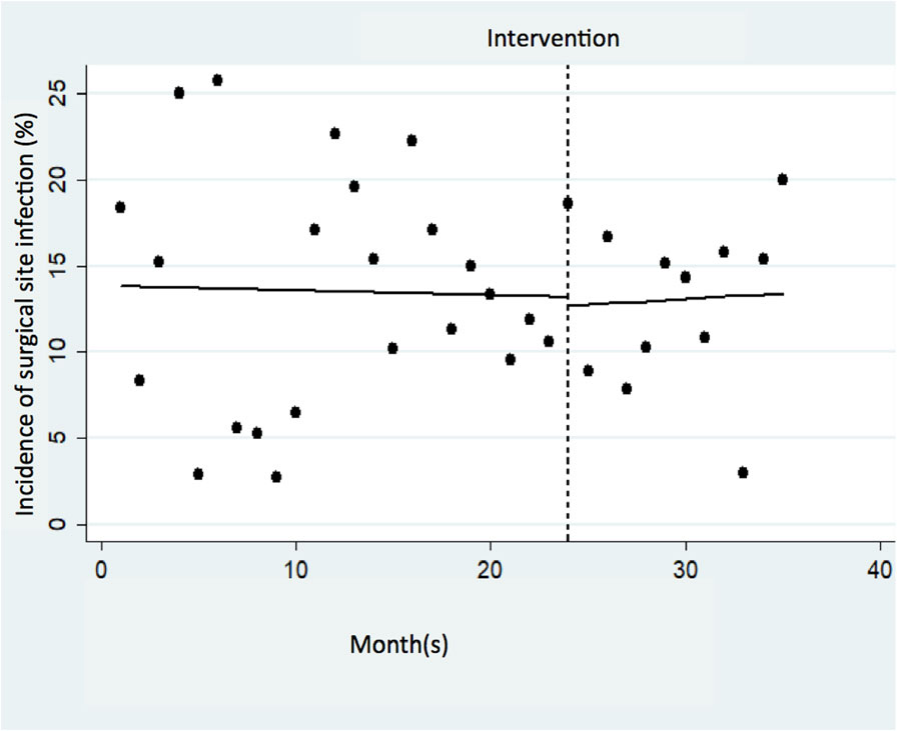
Interrupted time series analysis for assessing the impact of surgical antimicrobial prophylaxis intervention on the incidence of surgical site infection

Of the 1415 patients who underwent laparoscopic surgery during the study period, 1397 patients met the inclusion criteria (Fig. 1). Table 1 shows a summary of the patients’ baseline characteristics. In the intervention group, the median age was higher (68 years old vs. 66 years old; *p* = 0.04), and the proportion of selected comorbidities, such as cardiovascular diseases, was greater (7% vs. 3.9%; *p* = 0.01). Table 2 shows the changes in outcomes after implementing the intervention. The median interval between the time of completion of preoperative SAP and the time of surgical incision changed from 8 min to 26 min (*p* <  0.001), and the number of cases without a complete preoperative SAP prior to surgical incision decreased (16.8% vs. 1.8%; *p* <  0.001). However, the incidence of SSI did not significantly change between the pre-intervention and the intervention groups (13.8% vs. 13.2%; *p* = 0.80). The ITS model shown in Fig. 2 also revealed no statistically significant changes in SSI incidence after the intervention (intercept: *P* = 0.86; trend: *P* = 0.83). The model of factors associated with SSI development and the result of sensitivity analysis is shown in Appendix 2 (univariate analysis) and Table 3 (multivariate analysis), respectively. Overall, a trend towards older age and longer duration of surgery was observed in patients with SSI compared with those without SSI. The results of subgroup analysis for surgery type and SSI type are shown in Appendix 3. For all types of surgery, the number of cases without complete preoperative SAP prior to surgical incision decreased.

**Table 1:** Baseline characteristics of patients who underwent laparoscopic surgery in the study period (*n* = 1397)

	Pre-intervention (*n* = 942)	Intervention (*n* = 455)	*P* value
Age, median, (IQR) years	66 (52–75)	68 (55–76)	0.04
Male gender, n (%)	431 (56.4)	260 (57.1)	0.79
Co-morbidities, n (%)			
Diabetes mellitus	129 (13.7)	63 (13.8)	0.94
Chronic lung diseases	42 (4.5)	27 (5.9)	0.23
Cardiovascular diseases	37 (3.9)	32 (7.0)	0.01
Hypertension	333 (35.4)	180 (39.6)	0.14
Dyslipidemia	158 (16.8)	73 (16.0)	0.76
Current smoker, n (%)	446 (47.4)	224 (49.2)	0.51
ASA score, n (%)			
1 no disturbance	207 (22.0)	72 (15.8)	Ref.
2 mild disturbance	654 (69.4)	344 (75.6)	0.01
3 severe disturbance	79 (8.4)	37 (8.1)	0.22
4 life-threatening	2 (0.2)	2 (0.4)	0.30
Wound class, n (%)			
2 clean-contaminated	936 (99.4)	454 (99.8)	0.44
3 contaminated	6 (0.6)	1 (0.2)	0.44
Emergent operation, n (%)	152 (16.1)	70 (15.4)	0.72
Type of surgery, n (%)			
Gastrectomy	242 (25.7)	129 (28.4)	0.29
Cholecystectomy	213 (22.6)	94 (20.7)	0.41
Appendectomy	125 (13.3)	32 (7.0)	0.001
Colectomy	130 (13.8)	70 (15.4)	0.43
Proctectomy	231 (24.5)	130 (28.6)	0.11
Cholecystectomy and appendectomy	1 (0.1)	0 (0)	N/A
Operation time, median, (IQR) min	213 (133–286)	222 (145–322)	0.003

Abbreviations: *IQR* interquartile range, *ASA* American Society of Anesthesiologists, *N/A* not applicable

**Table 2:** Changes in outcomes after the intervention of SAP

	Pre-intervention (*n* = 942)	Intervention (*n* = 455)	*P* value
SAP, n (%)	940 (99.8)	455 (100)	
SAP drip infusion time, median, (IQR) min	16 (13–20)	23 (17–30)	< 0.001
Time from SAP completion to surgical incision, median, (IQR) min	8 (2–16)	26 (15–35)	< 0.001
Time windows, n (%)			
> 120 min	13 (1.4)	3 (0.7)	0.18
61–120 min	11 (1.2)	5 (1.1)	0.57
31–60 min	56 (5.9)	161 (35.4)	< 0.001
0–30 min	702 (74.5)	278 (61.1)	< 0.001
< 0 min	158 (16.8)	8 (1.8)	< 0.001
SSI, n (%)	130 (13.8)	60 (13.2)	0.80
Type of SSI, n (%)			
Superficial SSI	78 (8.3)	35 (7.7)	0.71
Deep SSI	0 (0)	4 (0.9)	N/A
Organ-space SSI	52 (5.5)	21 (4.6)	0.48

Abbreviations: *SAP* surgical antimicrobial prophylaxis, *IQR* interquartile range, *SSI* surgical site infection, *N/A* not applicable

**Table 3:** Univariate and multivariate analyses for potential risk factors for SSI

	Univariate analyses, OR (95% CI)	*P* value	Multivariate analyses, OR (95% CI)	*P* value
Age ≥ 65	1.44 (1.05–1.98)	0.02		
Male gender	0.82 (0.60–1.12)	0.21		
Diabetes mellitus	0.90 (0.59–1.40)	0.65		
Current smoker	1.61 (1.12–2.20)	0.003	1.66 (1.14–2.40)	0.01
ASA score ≥ 2	1.44 (0.95–2.19)	0.09		
Wound class 3 contaminated	2.57 (0.50–13.36)	0.26		
Emergent surgery	0.63 (0.39–1.01)	0.06		
Operation time ≥ 180 min	2.58 (1.79–3.71)	< 0.001	2.35 (1.56–3.54)	< 0.001
Time windows,				
31–60 min		Ref.		Ref.
< 0 min	0.67 (0.38–1.17)	0.16	0.78 (0.41–1.46)	0.43
0–30 min	0.58 (0.39–0.85)	0.01	0.65 (0.41–1.00)	0.052
> 60 min	0.72 (0.26–1.97)	0.52	1.14 (0.38–3.42)	0.81
Intervention status, “yes”	0.96 (0.69–1.33)	0.80	0.79 (0.54–1.15)	0.22

Abbreviations: *SSI* surgical site infection, *ASA* American Society of Anesthesiologists, *OR* odds ratio, *CI* confidence interval

## Discussion

In this study, we were able to prolong the interval between the completion of preoperative SAP and the first incision in laparoscopic surgery and to decrease the number of cases without complete preoperative SAP prior to surgical incision. The intervention successfully improved the quality of perioperative care in terms of SAP and likely increased awareness of the importance of SAP among health care workers. However, the intervention did not lead to a decrease in SSI incidence.

A number of studies have examined the optimal timing of preoperative SAP, with some studies showing that administration within 30 min prior to incision decreased the risk of postoperative infection [9, 10]. Other studies have reported that administration 30 to 60 min prior to incision was most effective in preventing SSI [11]. A recent systematic review demonstrated that the risk of SSI increased when preoperative SAP was administered 120 min before or after the first incision [12] while the WHO recommended that administration should be closer to the incision time (< 60 min) for antimicrobials with a short half-life, such as cephalosporins and penicillins [1]. At the moment, there are insufficient data to establish a more precise window [2, 13], and the present study was also unable to demonstrate the optimal timing for preoperative SAP.

A few previous studies showed that the risk of SSI increased when SAP was administered after incision [5, 12]. However, in the current study SSI incidence did not improve in the intervention period despite a decrease in the number of cases without a complete preoperative SAP prior surgical incision. Although the reasons for this result are unclear, several factors may have played a role. For instance, patients’ age in the intervention period was slightly higher than in the pre-intervention period. Previous studies showed an association between aging and increased risk of SSI [2, 14, 15]. Japan is one of the countries with an aging demographic [16]. Moreover, as seen in Table 2, the median duration of surgery in the intervention period was longer than in the pre-intervention period, an important risk factor for developing SSI [17]. Prolonged operative time in the intervention period might be explained by the patients’ age, which in turn was associated with a higher rate of comorbidities such as cardiovascular disease and a higher ASA score. Although SAP is one of the most important modifiable factors for reducing SSI, other, unmodifiable factors, including patient factors, might significantly contribute to the development SSI.

There are some limitations to our study. It is unclear whether the results would last beyond the brief, post-intervention period. Although patients’ baseline characteristics in both study periods were compared, the number of collected variables was limited, and potentially predisposing factors such as body mass index, steroid use, and nutritional status, which may have differed between the study periods, were not measured [2]. We also did not track information on the dosage of antimicrobials used in SAP or in additional antimicrobial administration for the longer operations. Lastly, as mentioned above, other modifiable measures (e.g. adequate skin preparation solutions, patient warming, and glycemic control) might have played an important role in preventing SSI.

## Conclusions

Although preoperative SAP is important, the timing of preoperative SAP within the 60-min window may be a minor detail for laparoscopic surgery. Moreover, other modifiable measures should be incorporated into comprehensive surgical quality improvement initiatives to reduce SSI.

## Appendix

**Appendix 1:** Recommended preoperative surgical antimicrobial prophylaxis at the study institution

Type of procedure	Agents	Dose	Redosing interval, hour
Gastrectomy	cefazolin	1–2 g	3–4
Cholecystectomy	cefazolin ampicillin/sulbactam	1-2 g 1.5-3 g	3–4 2–3
Appendectomy	cefazolin + metronidazole cefmetazole ampicillin/sulbactam	1-2 g + 500 mg 1-2 g 1.5-3 g	3–4 + 8 2–3 2–3
Colorectal surgery	cefazolin + metronidazole cefmetazole ampicillin/sulbactam	1-2 g + 500 mg 1-2 g 1.5-3 g	3–4 + 8 2–3 2–3

NOTE. For patients with β-lactam allergy and renal failure, infectious disease consultation is recommended

**Appendix 2:** Univariate analysis of factors associated with development of SSI

Variables	No SSI *n* = 1207	SSI *n* = 190	*P* value
Age, median, (IQR) years	66 (52–75)	70 (60–77)	0.001
< 65, n (%)	554 (45.9)	71 (37.4)	0.028
≥ 65, n (%)	653 (54.1)	119 (62.6)	
Male gender, n (%)	532 (44.1)	74 (39.0)	0.19
Co-morbidities, n (%)			
Diabetes mellitus	164 (13.6)	28 (14.7)	0.67
Chronic lung diseases	57 (4.7)	12 (6.3)	0.35
Cardiovascular diseases	59 (4.9)	10 (5.3)	0.83
Hypertension	442 (36.6)	71 (37.4)	0.84
Dyslipidemia	198 (16.4)	33 (17.4)	0.74
Current smoker, n (%)	560 (46.4)	110 (47.9)	0.003
ASA score, n (%)			
1 no disturbance	250 (20.7)	29 (15.3)	Ref.
2 mild disturbance	854 (70.8)	144 (75.8)	0.08
3 severe disturbance	99 (8.2)	17 (9.0)	0.23
4 life-threatening	4 (0.3)	0 (0)	N/A
Wound class, n (%)			
2 clean-contaminated	1202 (99.6)	188 (99.0)	0.25
3 contaminated	5 (0.4)	2 (1.1)	0.25
Emergent surgery, n (%)	200 (16.6)	22 (11.6)	0.080
Type of surgery, n (%)			
Cholecystectomy	291 (24.1)	16 (8.4)	Ref.
Gastrectomy	294 (24.4)	77 (40.5)	< 0.001
Appendectomy	140 (11.6)	17 (9.0)	0.029
Colectomy	175 (14.5)	25 (13.2)	0.004
Proctectomy	306 (25.4)	55 (29.0)	< 0.001
Cholecystectomy and appendectomy	1 (0.1)	0 (0)	N/A
Operation time, median, (IQR) min	205 (131–285)	273 (197–337)	< 0.001
< 180, n (%)	502 (41.6)	42 (22.1)	
≥ 180, n (%)	705 (58.4)	148 (77.9)	< 0.001
SAP drip infusion time, median, (IQR) min	18 (14–23)	18 (14–24)	0.97
Time from SAP completion to surgical incision, median, (IQR) min	12 (4–25)	15 (3–31)	0.37
31–60 min	175 (14.5)	42 (22.2)	Ref.
< 0 min	143 (11.9)	23 (12.2)	0.16
0–30 min	861 (71.4)	119 (63.0)	0.005
> 60 min	26 (2.2)	6 (3.2)	0.52
Intervention status, “yes”	395 (32.7)	60 (31.6)	0.75

NOTE. *SSI* surgical site infection, *IQR* interquartile range, *ASA* American Society of Anesthesiologists, *SAP* surgical antimicrobial prophylaxis, *N/A* not applicable

**Appendix 3:** Subgroup analysis of different types of SSI

	No SSI (*n* = 1207)	Superficial SSI (*n* = 113)	Deep and organ space SSI (*n* = 77)	*P* value	
				No SSI vs superficial SSI	No SSI vs deep and organ space SSI
Age, median, (IQR) years	66 (52–75)	67 (53–76)	72 (64–78)	0.49	< 0.001
< 65, n (%)	554 (45.9)	50 (44.3)	21 (27.3)	0.74	0.018
≥ 65, n (%)	653 (54.1)	63 (55.7)	56 (72.7)		
Male gender, n (%)	532 (44.1)	57 (50.4)	17 (22.1)	0.19	< 0.001
Co-morbidities, n (%)					
Diabetes mellitus	164 (13.6)	13 (11.5)	15 (19.5)	0.53	0.15
Chronic lung diseases	57 (4.7)	6 (5.3)	6 (7.8)	0.78	0.23
Cardiovascular diseases	59 (4.9)	7 (6.2)	3 (3.9)	0.54	0.69
Hypertension	442 (36.6)	38 (33.6)	33 (42.9)	0.53	0.27
Dyslipidemia	198 (16.4)	19 (16.8)	14 (18.2)	0.91	0.68
Current smoker, n (%)	560 (46.4)	58 (51.3)	52 (67.5)	0.32	< 0.001
ASA score, n (%)					
1 (no disturbance)	250 (20.7)	22 (19.5)	7 (9.1)	Ref.	Ref.
2 (mild disturbance)	854 (70.8)	82 (72.6)	62 (80.5)	0.73	0.02
3 (severe disturbance)	99 (8.2)	9 (8.0)	8 (10.4)	0.94	0.05
4 (life-threatening)	4 (0.3)	0 (0)	0 (0)	N/A	N/A
Wound class, n (%)					
2 clean-contaminated	1202 (99.6)	112 (99.1)	76 (98.7)	0.48	0.27
3 contaminated	5 (0.4)	1 (0.9)	1 (1.3)	0.48	0.27
Emergent surgery, n (%)	200 (16.6)	16 (14.2)	6 (7.8)	0.51	0.04
Type of surgery, n (%)					
Cholecystectomy	291 (24.1)	11 (9.7)	5 (6.5)	Ref.	Ref.
Gastrectomy	294 (24.4)	28 (24.8)	49 (63.6)	0.011	< 0.001
Appendectomy	140 (11.6)	14 (12.4)	3 (3.9)	0.019	0.77
Colectomy	175 (14.5)	16 (14.2)	9 (11.7)	0.028	0.05
Proctectomy	306 (25.4)	44 (38.9)	11 (14.3)	< 0.001	0.18
Cholecystectomy and appendectomy	1 (0.1)	0 (0)	0 (0)	N/A	N/A
Operation time, median, (IQR) min	205 (131–285)	229 (164–296)	325 (271–399)	0.015	< 0.001
< 180, n (%)	502 (41.6)	33 (29.2)	9 (11.7)	0.01	< 0.001
≥ 180, n (%)	705 (58.4)	80 (70.8)	68 (88.3)		
SAP drip infusion time, median, (IQR) min	18 (14–23)	18 (14–25)	17 (13–22)	0.52	0.46
Time from SAP completion to surgical incision, median, (IQR) min	12 (4–25)	14 (2–31)	17 (6–29)	0.39	0.69
Time windows, n (%)					
31–60 min	175 (14.5)	24 (21.2)	18 (23.4)	Ref.	Ref.
< 0 min	143 (11.9)	18 (15.9)	5 (6.5)	0.80	0.04
0–30 min	861 (71.4)	65 (57.5)	54 (70.1)	0.02	0.08
> 60 min	26 (2.2)	6 (5.3)	0 (0)	0.30	N/A
Intervention status, “yes”	395 (32.7)	35 (31.0)	25 (32.5)	0.70	0.83

NOTE. *SSI* surgical site infection, *IQR* interquartile range, *ASA* American Society of Anesthesiologists, *SAP* surgical antimicrobial prophylaxis, *N/A* not applicable

## Abbreviations

ASA: American Society of Anesthesiologists
CDC: Center for Disease Control and Prevention
SAP: Surgical antimicrobial prophylaxis
SSI: Surgical site infection
WHO: World Health Organization
